# Taxonomy of *Macromotettixoides* with the description of a new species (Tetrigidae, Metrodorinae)

**DOI:** 10.3897/zookeys.645.9055

**Published:** 2017-01-12

**Authors:** Ling-Sheng Zha, Feng-Ming Yu, Saranyaphat Boonmee, Prapassorn D. Eungwanichayapant, Ting-Chi Wen

**Affiliations:** 1 The Engineering and Research Center for Southwest Bio-Pharmaceutical Resources of National Education Ministry of China, Guizhou University, Guiyang 550025, China; 2 School of Life Sciences, Huaibei Normal University, Huaibei 235000, China; 3 Institute of Excellence in Fungal Research, and School of Science, Mae Fah Luang University, Chiang Rai 57100, Thailand

**Keywords:** China, determination key, ecology, habit, Hyboella, Karst Region, Orthoptera, revision, Tetrigoidea

## Abstract

Descriptions of the flying organs and generic characteristics of the genus *Macromotettixoides* Zheng, Wei & Jiang are currently imprecise. *Macromotettixoides* is reviewed and compared with allied genera. A re-description is undertaken and a determination key is provided to *Macromotettixoides*. *Macromotettixoides parvula* Zha & Wen, **sp. n.** from the Guizhou Karst Region, China, is described and illustrated with photographs. Observations on the ecology and habits of the new species are recorded. Four current species of *Hyboella* Hancock are transferred to *Macromotettixoides*. Variations of the flying organs and tegminal sinus in the Tetrigidae are discussed, which will help to describe them accurately.

## Introduction

The originally monotypic genus *Macromotettixoides* (Orthoptera: Tetrigidae: Metrodorinae) was erected by Zheng et al. in 2005 with *Macromotettixoides jiuwanshanensis* Zheng, Wei & Jiang, 2005 as its type species. Deng et al. (2014) gave a systematic study of this genus, which dealt with eight known species. Simultaneously Zheng (2013a) introduced another new species, *Macromotettixoides wuyishana* Zheng, 2013. Recently Deng (2016) in his dissertation transfered *Apterotettix lativertex* Zheng, Ou & Zhang, 2012 to this genus and assigned a new name, *Macromotettixoides longling* (Zheng, Ou & Zhang, 2012) for it, because the epithet ‘*lativertex*’ had already been used in this genus, also described another new species, *Macromotettixoides jinggangshanensis* Deng, 2016. As a result, *Macromotettixoides* currently includes 11 species, which are all distributed in China.

Based on absent tegmen (accordingly tegminal sinus is absent or inconspicuous), Zheng et al. (2005) erected *Macromotettixoides* to distinguish from *Macromotettix* Günther, 1939; and for the reason of shortened hind process and with broad and arcuate apex, Zheng et al. (2012) erected *Pseudomacromotettix* Zheng, Li & Lin, 2012 to separate from *Macromotettixoides*. To these three allied genera there are some problems we are facing: 1) descriptions to both flying organs and tegminal sinus are ambiguous; 2) descriptions of generic characteristics of *Macromotettixoides* are vague (Deng et al. 2014, Deng 2016); and 3) features of partial species of *Hyboella* Hancock, 1915 are also in accord with *Macromotettixoides*, but the researches above seldom dealt with *Hyboella*.

In this study *Macromotettixoides* is examined and compared it with allied genera. At the same time *Macromotettixoides parvula* Zha & Wen, sp. n., from Guizhou Karst Region, China, is described and illustrated with photographs. Some aspects of ecology and observations of habits of the new species are also recorded.

## Material and methods

Specimens were photographed using a stereo microscope (Olympus Corporation, SZX16, Tokyo, Japan), ecological pictures were photographed using a Nikon Coolpix P520 camera. Morphological terminology and measurement landmarks follow Zheng (2005) and Tumbrinck (2014). Measurements are given in millimeters (mm). Type specimens are deposited in the Specimen Room of the School of Life Sciences, Huaibei Normal University, Huaibei, Anhui Province, China.

## Taxonomy

### Macromotettixoides

Taxon classificationAnimaliaOrthopteraTetrigidae

Zheng, Wei & Jiang, 2005

Macromotettixoides Zheng, Wei & Jiang 2005: 366; Zheng 2005: 176; Deng et al. 2007: 160, 2014: 548; Deng 2011: 543, 2016: 155.

#### Type species.

*Macromotettixoides jiuwanshanensis* Zheng, Wei & Jiang, 2005 by original designation

#### Redescription.

Size small and stout. Vertex nearly at the same or slightly below the level of anterior margin of pronotum, and decidedly wider than width of one eye; longitudinal furrow (instead of scutellum in most genera of Cladonotinae) relatively shallow, equal to or wider than diameter of scapus (similar to Cladonotinae); antennae filiform, inserted between or below lower margin of eyes. Pronotum roof-like or nearly at the same level; median carina conspicuous, sometime weakly, but not strongly lamellate; hind process short, not surpassing apex of hind femur, apex acute or acutely rounded; posterior angle of lateral lobe turning outwards (differ from Tettiginae (directed downwards and contiguous to the body)), apex truncated or roundly truncated (differ from Scelimeninae (directed sideward as an acute triangle process or a long acute spine)); ventral sinus present, tegminal sinus absent or very inconspicuous; external lateral carina surpassing middle of lower margin of pronotum. Flying organs abbreviated: tegmina invisible; hind wings invisible in most species, visible but never reaching middle of hind process in few species. Female ovipositor narrow and long.

#### Distribution.

China (Fujian, Guangdong, Guangxi, Guizhou, Hainan, Hubei, Hunan, Jiangxi, Sichuan, Yunnan, Taiwan).

#### Key to species of *Macromotettixoides* (16 species)

**Table:** 

1	Hind wings very small and hidden beneath pronotum, invisible or barely visible	**2**
–	Hind wings slightly elongate and nearly reaching middle of hind femur, visible	**15**
2	Pronotum flattened, nearly at the same level	**3**
–	Pronotum, in lateral view, distinctly roof-like	**5**
3	Anterior margin of pronotum broadly arcuate forward; humeral angles also broadly arcuate (Hainan)	***Macromotettixoides hainanensis* (Liang, 2002), comb. n.**
–	Anterior margin of pronotum truncated; humeral angles obtuse angled	**4**
4	Vertex 1.4 times as wide as one eye; prozonal carinae contracted backward; hind process reaching middle of hind femur (Taiwan)	***Macromotettixoides taiwanensis* (Liang, 2000), comb. n.**
–	Vertex 2.0 times as wide as one eye; prozonal carinae parallel; hind process reaching knee of hind femur (Guizhou)	***Macromotettixoides parvula* sp. n.**
5	Anterior margin of pronotum obtusely angled forward	**6**
–	Anterior margin of pronotum truncated	**10**
6	Upper margin of pronotum wholly arcuate in lateral view; hind process reaching apex of hind femur	**7**
–	Upper margin of pronotum, in lateral view, arcuate only before humeral angles while straight or undulated behind humeral angles; hind process not reaching apex of hind femur	**9**
7	Vertex 3.0 times as wide as one eye, anterior margin obtusely angled (Fujian)	***Macromotettixoides wuyishana* Zheng, 2013a**
–	Vertex 2.1-2.3 times as wide as one eye, anterior margin arcuate	**8**
8	Vertex together with frontal costa right angled; humeral angles obtusely angled, interhumeral carina absent; middle of posterior margin of female subgenital plate with a triangular protrusion (Guangxi)	***Macromotettixoides jiuwanshanensis* Zheng et al., 2005**
–	Vertex together with frontal costa rounded; humeral angles absent, paired interhumeral carinae presented; posterior margin of female subgenital plate three-tooth-like (Jiangxi)	***Macromotettixoides jinggangshanensis* Deng, 2016**
9	Longitudinal furrow between antennal grooves 1.6 times as wide as diameter of scapus; pronotal disc with many net-like wrinkles; humeral angles indistinct; lower margins of fore and mid femora a little undulate (Guangxi)	***Macromotettixoides lativertex* Deng et al., 2014**
–	Longitudinal furrow between antennal grooves as wide as diameter of scapus; pronotal disc smooth; humeral angles arcuate; lower margins of fore and mid femora straight (Jiangxi)	***Macromotettixoides brachynota* Zheng & Shi, 2009**
10	Lower margins of fore and mid femora undulated	**11**
–	Lower margins of fore and mid femora straight	**12**
11	Antenna inserted below lower margin of eyes; prozonal carinae parallel; apex of hind process narrow (Sichuan)	***Macromotettixoides undulatifemura* Deng et al., 2012**
–	Antenna inserted between lower margin of eyes; prozonal carinae contracted backward; apex of hind process relatively wide, concave in the middle (Yunnan)	***Macromotettixoides curvimarginus* (Zheng & Xu, 2010), comb. n.**
12	Vertex 2.0-2.14 times as wide as one eye; interhumeral carina absent	**13**
–	Vertex 1.3-1.6 times as wide as one eye; paired interhumeral carinae presented	**14**
13	In lateral view upper margin of pronotum before shoulders strongly arcuate; humeral angles obtusely rounded (Hubei)	***Macromotettixoides wufengensis* Zheng et al., 2009**
–	In lateral view upper margin of pronotum straight; humeral broadly arcuate (Hunan)	***Macromotettixoides badagongshanensis* (Zheng, 2013b), comb. n.**
14	Vertex 1.3 times as wide as one eye; antenna inserted below lower margin of eyes; in lateral view upper margin of pronotum strongly arcuate before humeral angles while straight behind humeral angles (Fujian)	***Macromotettixoides zhengi* Deng, 2011**
–	Vertex 1.6 times as wide as one eye; antenna inserted between lower margin of eyes; in lateral view upper margin of pronotum nearly straight (Yunnan)	***Macromotettixoides longling* (Zheng et al., 2012)**
15	Vertex 2.0 times as wide as one eye; hind process reaching middle of hind femur; lower margin of mid femur undulate (Yunnan)	***Macromotettixoides cliva* Zheng et al., 2006**
–	Vertex 1.5 times as wide as one eye; hind process reaching two-thirds of hind femur; lower margin of mid femur straight (Guizhou)	***Macromotettixoides aelytra* (Zheng et al., 2002)**, nymph*

*Note: according to descriptions (antegenicular denticle and genicular denticle have not been separated) and drawings of Zheng et al. (2002) and Zheng (2005), the type specimen (only one female) of *Macromotettixoides aelytra* (Zheng, Li & Shi, 2002) (synonym: *Hyboella aelytra* Zheng, Li & Shi, 2002 (Zheng et al. 2006)) should be a nymph. Validity of *Macromotettixoides aelytra* requires more material to confirm its characters, and herein we temporarily place the species in the key.

### Macromotettixoides parvula

Taxon classificationAnimaliaOrthopteraTetrigidae

Zha & Wen sp. n.

http://zoobank.org/F2EFC917-2D8A-4A9A-89E8-DD3A3EA6861D

Figs 1 , 2

#### Diagnosis.

With extremely small size, *Macromotettixoides parvula* sp. n. can easily be separated from other species of the genus. Other differences are listed in the key to species of *Macromotettixoides*.

#### Description.

**Female.** Body size extremely small.

*Head*. Face and vertex rough, covered with large and small granules. Vertex nearly at the same level but uneven, 2.0 times as wide as one eye, a little contracted forward, protruding forward and slightly surpassing anterior margin of eyes; anterior margin broadly arcuate and depressed, anterior part of lateral carina distinctly folded upward and reaching top of eyes; medial carina distinct and erected in anterior half, but absent in posterior half; paired fossulae deep, behind fossulae vertex slightly elevated on both sides (Fig. 2b). In lateral view face slightly oblique, fastigium (vertex together with frontal costa) rounded and protruding forward; fascial carinae smooth, between lateral ocelli concave, between antennal grooves widely and obtusely triangular forward (Fig. 2d); in frontal view fascial carinae diverged in the middle of inner margin of eyes, longitudinal furrow wide and shallow and nearly forming into a scutellum, between antennal grooves 1.5 times as wide as diameter of scapus (Fig. 2a). Antenna filiform and short, 17-segmented, inserted decidedly below lower margin of eyes, segment 11 longest, 5.0 times as long as wide (Fig. 2a, d). Eyes globose and protruding, over level of anterior margin of pronotum, lateral ocelli placed at lower one third of inner margin of eyes (Fig. 2a).

**Figure 1.**
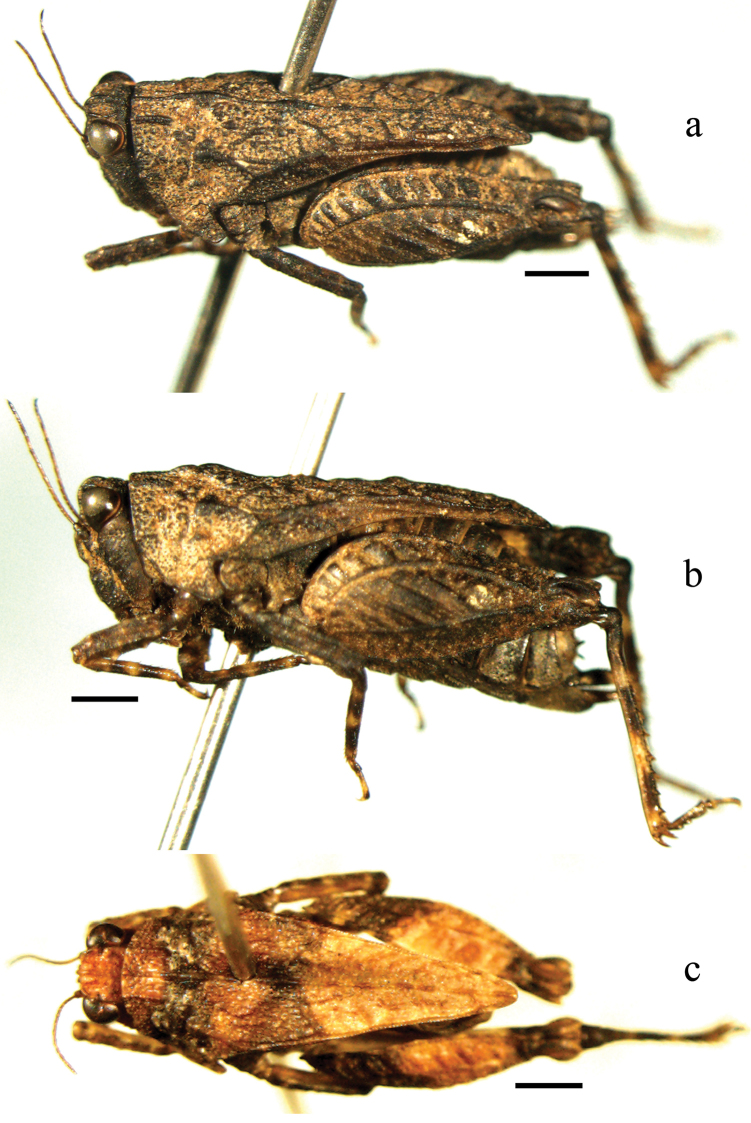
*Macromotettixoides parvula* sp. n. **a** oblique-lateral view of female body **b** lateral view of female body **c** dorsal view of male body. Scale bars 1.0 mm.

**Figure 2.**
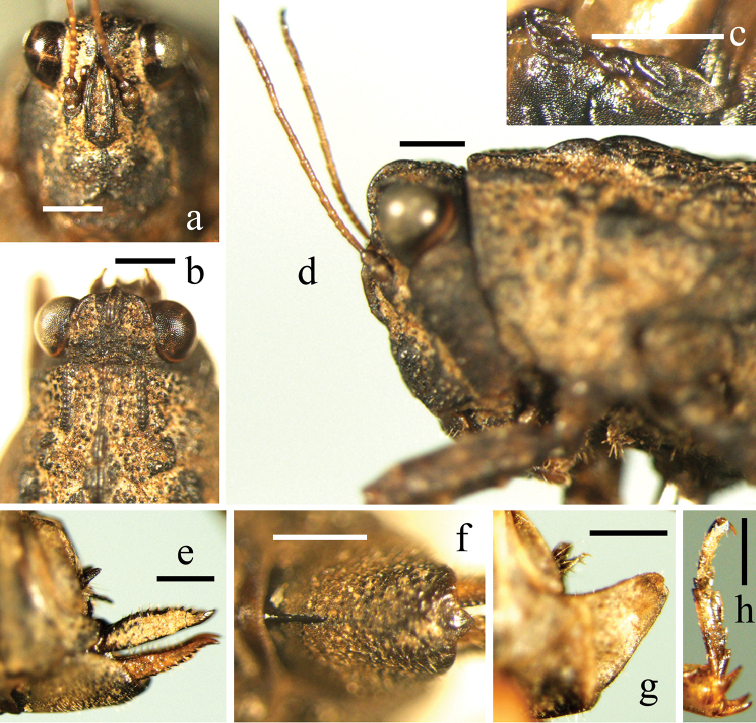
*Macromotettixoides parvula* sp. n. **a** frontal view of female head **b** dorsal view of female head **c** left-lateral view of female tegmen and hind wing after uncovering the pronotum **d** lateral view of female head and anterior pronotum **e** lateral view of female ovipositor and subgenital plate **f** ventral view of female subgenital plate **g** lateral view of male subgenital plate **h** left-lateral view of female hind tarsus. Scale bars 0.5 mm.

*Thorax*. Pronotum disc very coarse, covered with big and small granules and many thick and net-like wrinkles (Fig. 1). Anterior margin truncated and wide, paired extralateral carinae indistinct (Fig. 2b); pronotum in the center between posterior sulcus and humeral angles slightly elevated, otherwise nearly at the same level. Median carina entire and thick, between sulci distinctly elevated with swollen base, behind humeral angles with a protrusion, the posterior protrusions lower and indistinct; in lateral view upper margin undulate, the first sinusoidal wave occur between sulci, longest and highest, in the middle with a deep concavity, followed by the second wave behind humeral angles, the posterior waves low and nearly at the same height. Prozonal carinae conspicuous, thick, erected and parallel (Fig. 2b); humeral angles obtuse angled and low, pronotum disc behind humeral angles covered with many reticular wrinkles; hind process reaching knees (three males and two females) or barely surpass apex of hind femur (one female), apex sharp-rounded; external lateral carina straight, distinctly surpassing middle of lower margin and reaching two-thirds of hind femur, folded upward indistinctly behind humeral angles; lateral carina of pronotum before apex curved inward; posterior angle of lateral lobe of pronotum extending turning outwards, margin smooth, apex truncated and anterior margin of apex rounded; posterior margin of each lateral lobe with one concavity. Tegmina and wings extremely degenerated, long and oval, apices acute, wing distinctly larger than tegmen, both hidden beneath pronotum and invisible (Fig. 2c). Margins of all femora serrate except base of upper margin of hind femur, upper margins of fore and mid femora nearly straight while lower margins with two teeth (basal and middle) each; hind femur stout, 2.3 times as long as wide, upper margin before antegenicular denticle with a small tooth, other teeth on upper and lower margins indistinct; antegenicular denticle slightly isolated, low, apex or nearly right angled or a little sharp, genicular denticle finger-like, extending backward and apex obtuse; margins of fore and mid tibiae straight; two inner margins of hind tibia serrate, terminal part slightly wider than basal part, outer/inner side with 6-7/4-6 spines; first segment of hind tarsus 1.35 times as long as second plus third, first and second pulvilli small and apices sharp, third pulvillus large and apex obtuse (Fig. 2h).

*Abdomen*. Ovipositor: upper valva about 4.0 times as long as wide, upper margin arcuate, sub-base widest, in the middle slightly distorted inward, then slightly turn outward and at last inward again; outer margins of upper and lower valvae with saw-like teeth, but base of upper valva smooth (Fig. 2e). Subgenital plate: length nearly equal to width, median carina distinct in anterior part, posterior margin nearly truncated and in the middle triangularly protruding which is slightly folded inward (Fig. 2f).

*Coloration*. Body dark or dark brown (Fig. 1). Antennae brown, color of terminal 3-5 segments dark, color of the two segments of before and after the longest segment a little light (Fig. 2d). Sometimes both the posterior part of pronotum and the posterior part of outer side of hind femur brown. All tibiae with three yellowish brown rings each, but basal and middle rings of hind tibia large. More or less, infrascapular area, teeth on lower margins of fore and mid femora, upper and lower margin of hind femur, and outer sides of all femora maculated with yellowish brown.

**Male.** Slightly smaller than female (Fig. 1). Vertex also 2.0 times as wide as one eye; antenna 16 segmented, segment 10 longest. Subgenital plate short cone-shaped, apex nearly truncated, upper apex bifurcate and forming into two obtuse and very short teeth (Fig. 2g). Other characters same as female.

#### Measurements.

Length of body ♂5.8–6.2 mm, ♀7.5–8.3 mm; length of pronotum ♂5.8–6.0 mm, ♀6.3–7.0 mm; length of hind femur ♂4.1–4.3 mm, ♀4.2–4.5 mm; length of antenna ♂, ♀2.6–2.8 mm.

#### Type material.

Holotype female, China, Guizhou, Leishan, Leigongshan Mountain, N26°22'18.25", E108°11'28.06", 1430 m alt, 2 Aug. 2016, collected by Lingsheng ZHA. Paratypes: three males and two females, Leigongshan Mountain, 1300–1600 m alt, 1–3 Aug. 2016, collected by Lingsheng ZHA.

#### Ecology and habits.

**Figure 3.**
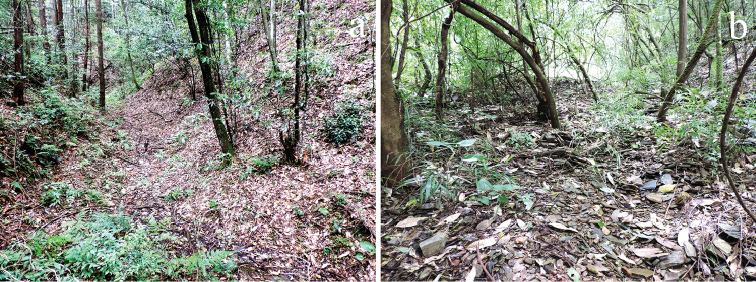
Habitat environment of *Macromotettixoides parvula* sp. n. **a** a gully in a broad-leaved forest **b** border of a stream. Pictures were photographed by Lingsheng Zha in China, Guizhou, Leishan, Leigongshan Mountain, 2 Aug 2016.

Specimens of *Macromotettixoides parvula* sp. n. were collected and observed among low and sparse shrubs with fall-leaf layers in gullies, slopes and a dry stream bed in humid rainforests of Karst Region (Fig. 3). They are very small and not easy to find; they move quickly and they like to jump into shrubs when being disturbed. They mainly feed on humus. We infer their adults may prefer to stay in sandy soil, because body surfaces of most specimens are covered tightly by sandy soil (Zha et al. 2016a, fig. 1a, b).

#### Etymology.

The new species epithet ‘*parvula*’ means body size is extremely small.

#### Distribution.

China (Guizhou). Only found in Leigongshan Mountain (Leishan County).

## Discussion

### Flying organs and tegminal sinus in Tetrigidae

According to Zha et al. (2016b), hind wings of Tetrigidae can be divided into four types: ‘normal’ (developed, nearly reach apex of hind process or more), ‘abbreviated’ (never reach middle of hind process, but distinctly longer than tegmen), ‘vestigial’ (shorter than tegmen) and ‘apterous’ (absent, degenerated completely). In *Macromotettixoides* and *Pseudomacromotettix*, we believe their tegmina are presented and their hind wings belong to the ‘abbreviated’ (Fig. 2c) or ‘vestigial’ type, so using ‘absent’ to describe their small flying organs is exactly not suitable if not uncovering pronota. In this report, we use ‘invisible’ to replace ‘absent’/‘wanting’ for describing tegmen and wing. To some species of ‘abbreviated’ or ‘vestigial’ type, a little visible parts of their fly organs may vary distinctly even become invisible among the same species, which should not be considered as a valuable taxonomic character (Zha et al. 2016b). We also believe tegminal sinus varies according to tegmen strictly during evolution. In other words, normal tegmen means that the tegminal sinus is conspicuous; on the contrary, invisible or a little visible tegmen has determined that the tegminal sinus is absent or shallow.

### Relationships between *Macromotettixoides* and its allied genera

In order to clarify relationship between *Macromotettixoides* and its allied genera, we summarize their main differences, based on their known species, as in Table 1. Undoubtedly, *Macromotettixoides* is most similar to *Pseudomacromotettix* and *Macromotettix* (see Introduction and Table 1).

**Table 1.** Main differences between *Macromotettixoides* and its allied genera.

Characters Genera	Vertex and anterior margin of pronotum		Antenna inserted above, between or below lower margin of eye			Tegminal sinus		External lateral carina reaching lower margin of pronotum	
	Nearly at the same level	Vertex distinctly higher	Lower 1/3 of inner margin	Between or slightly below	Far away below	Absent or inconspicuous	Presented	Middle or more	Before middle
*Pseudomacromotettix*	√			√		√		√	
*Macromotettixoides*	√			√		√		√	
*Macromotettix*	√			√			√	√	
*Hyboella*	√		√	√		√?	√	√	√
*Cotysoides*	√		√				√		√
*Bolivaritettix*	√			√			√		√
*Mazarredia*		√		√			√		√
*Xistrella*		√			√		√		√

According to Hancock (1915), Günther (1939), Zheng (2005) and Deng (2016), the typical characteristic of *Hyboella* is a pronotum distinctly humpbacked and elevated before the shoulders while depressed and flattened behind shoulders. This characteristic can separate *Hyboella* from *Pseudomacromotettix*, *Macromotettix*, *Cotysoides* Zheng & Jiang, 2000, and *Bolivaritettix* Günther, 1939 where their pronota are wholly roof-like or nearly at the same level. Notably, partial species of *Macromotettixoides* also have this similar character (see the key)! The type species of *Hyboella*, *Hyboella tentata* Hancock, 1915, not only possesses this typical characteristic, but also has a conspicuous tegminal sinus and normal flying organs. Therefore, only depending upon the conspicuous tegminal sinus and normal flying organs can one separate *Hyboella* from *Macromotettixoides* (Table 1). In light of this, we suggest that species currently placed in *Hyboella* whose tegminal sinus is absent (accordingly, the tegmen is invisible), and also whose hind wing is ‘abbreviated’ or ‘vestigial’, should be transferred to *Macromotettixoides*. Just as in species of *Macromotettixoides*, we also believe no ‘apterous’ species occurr in *Hyboella*.

Herein we transfer the related Chinese species of *Hyboella* whose tegminal sinuses are all absent (their flying organs are all invisible); also their pronota do not meet the typical characteristic of *Hyboella* (wholly roof-like or flattened), into *Macromotettixoides* as follows:

*Macromotettixoides badagongshanensis* (Zheng, 2013b), comb. n. = *Hyboella badagongshanensis* Zheng, 2013b;

*Macromotettixoides curvimarginus* (Zheng & Xu, 2010), comb. n. = *Hyboella curvimarginus* Zheng & Xu, 2010;

*Macromotettixoides hainanensis* (Liang, 2002), comb. n. = *Hyboella hainanensis* Liang, 2002;

*Macromotettixoides taiwanensis* (Liang, 2000), comb. n. = *Hyboella taiwanensis* Liang, 2000.

## Supplementary Material

XML Treatment for Macromotettixoides

XML Treatment for Macromotettixoides parvula
